# Waterborne Isolates of *Campylobacter jejuni* Are Able to Develop Aerotolerance, Survive Exposure to Low Temperature, and Interact With *Acanthamoeba polyphaga*

**DOI:** 10.3389/fmicb.2021.730858

**Published:** 2021-10-27

**Authors:** Ekaterina Shagieva, Katerina Demnerova, Hana Michova

**Affiliations:** Laboratory of Food Microbiology, Department of Biochemistry and Microbiology, University of Chemistry and Technology, Prague, Czechia

**Keywords:** *Campylobacter je* *juni*, water isolates, oxidative stress, low temperature, aerotolerance, *Acanthamoeba polyphaga*

## Abstract

*Campylobacter jejuni* is regarded as the leading cause of bacterial gastroenteritis around the world. Even though it is generally considered to be a sensitive microaerobic pathogen, it is able to survive in the environment outside of the intestinal tract of the host. This study aimed to assess the impact of selected environmental parameters on the survival of 14 *C. jejuni* isolates of different origins, including 12 water isolates. The isolates were tested for their antibiotic resistance, their ability to survive at low temperature (7°C), develop aerotolerance, and to interact with the potential protozoan host *Acanthamoeba polyphaga*. The antibiotic susceptibility was determined by standard disk diffusion according to EUCAST. Out of the 14 isolates, 8 were resistant to ciprofloxacin (CIP) and 5 to tetracycline (TET), while only one isolate was resistant to erythromycin (ERY). Five isolates were resistant to two different antibiotic classes. Tetracycline resistance was only observed in isolates isolated from wastewater and a clinical sample. Further, the isolates were tested for their survival at 7°C under both aerobic and microaerobic conditions using standard culture methods. The results showed that under microaerobic conditions, all isolates maintained their cultivability for 4 weeks without a significant decrease in the numbers of bacteria and variation between the isolates. However, significant differences were observed under aerobic conditions (AC). The incubation led to a decrease in the number of cultivable cells, with complete loss of cultivability after 2 weeks (one water isolate), 3 weeks (7 isolates), or 4 weeks of incubation (6 isolates). Further, all isolates were studied for their ability to develop aerotolerance by repetitive subcultivation under microaerobic and subsequently AC. Surprisingly, all isolates were able to adapt and grow under AC. As the last step, 5 isolates were selected to evaluate a potential protective effect provided by *A. polyphaga*. The cocultivation of isolates with the amoeba resulted in the survival of about 40% of cells treated with an otherwise lethal dose of gentamicin. In summary, *C. jejuni* is able to adapt and survive in a potentially detrimental environment for a prolonged period of time, which emphasizes the role of the environmental transmission route in the spread of campylobacteriosis.

## Introduction

*Campylobacter jejuni* is one of the main bacterial causes of gastroenteritis in Europe, with about 300,000 reported cases per year (EFSA, 2021). The infection by this pathogen usually leads to the development of gastroenteritis (campylobacteriosis) with fever, abdominal pain, dysentery, and nausea. In most cases, the illness is self-limiting with a duration varying from a few days to weeks (Horrocks et al., 2009; Silva et al., 2011). In some cases, such as in immunocompromised people, *C. jejuni* can be a precursor of autoimmune diseases, such as Guillain-Barré syndrome, reactive arthritis, irritable bowel syndrome, and inflammatory bowel disease (Rees et al., 1995; Ang et al., 2001; Ternhag et al., 2008). As the main reservoir of *C. jejuni* is the gastrointestinal tract of wild and domestic animals, especially birds, campylobacteriosis is mainly associated with the consumption of raw or undercooked poultry products (Kaakoush et al., 2015; Skarp et al., 2016). However, more and more data have been published showing that non-food sources, such as drinking and surface water, can participate in the dissemination of the disease (O’Reilly et al., 2007; Uhlmann et al., 2009; Whiley et al., 2013; Guzman-Herrador et al., 2015; Moreira and Bondelind, 2017; Ahmed et al., 2020; Hyllestad et al., 2020; Kuhn et al., 2020; Tzani et al., 2020). *C. jejuni* cells were also isolated from rivers (Khan et al., 2014), agricultural waters (Mulder et al., 2020), lakes (Kemp et al., 2005), children’s pool waters (Sawabe et al., 2016), beach sand samples (Yamahara et al., 2012) and playgrounds (French et al., 2009). Fecally contaminated drinking water has been associated with several *Campylobacter* outbreaks in Sweden (Martin et al., 2006), Finland (Miettinen et al., 2001; Hänninen et al., 2003), England (Smith et al., 2006), the United States (Pedati et al., 2019), Norway (Paruch et al., 2020), New Zealand (Bartholomew et al., 2014; Gilpin et al., 2020) and Denmark (Kuhn et al., 2017). Most of the outbreaks have been attributed to the consumption of untreated groundwater.

*Campylobacter jejuni* is unique among foodborne pathogens in its obligate microaerophilic nature and narrow range of growth temperatures, with a minimum growth temperature ranging from 31 to 36°C (Hazeleger et al., 1998; Davis and DiRita, 2008), and optimum at 42°C. Some studies suggest that despite its strict growth requirements, *C. jejuni* has the ability to survive under conditions that otherwise inhibit its growth, such as an atmospheric concentration of oxygen. Exposure to an aerobic atmosphere leads to oxidative stress caused by the formation of reactive oxygen species (ROS), which among other things destroy numerous Fe-S proteins of *C. jejuni*. Although a limited number of ROS detoxification enzymes are expressed in *C. jejuni* (Atack and Kelly, 2009), a few previously isolated isolates were reported to be aerotolerant (Oh et al., 2015a; Bronnec et al., 2016; Karki et al., 2018). They can effectively survive under aerobic conditions (AC) and therefore pose a serious risk of food contamination and human infection. The hyper-aerotolerant isolates (those remaining viable after 24 h of aerobic incubation) of *C. jejuni* were so far isolated from raw poultry meat, a duck slaughterhouse, human clinical samples, and dairy products (Rodrigues et al., 2015; Guk et al., 2019; Kiatsomphob et al., 2019; Mouftah et al., 2021). Several studies suggested that *C. jejuni* also has the potential to survive under non-permissive temperature conditions. It is known that cells of *C. jejuni* exposed to ambient temperature remained viable for a few days, while cells cultivated at 4°C survived for several weeks (Terzieva and McFeters, 1991; Buswell et al., 1998), or even months when transformed into a non-cultivable coccoid form (Rollins and Colwell, 1986; Jones et al., 1991; Hazeleger et al., 1995).

The survival of *Campylobacter* in water is influenced by many factors, such as the concentration of dissolved oxygen (Buswell et al., 1998), presence of ammonium, chloride ions and phosphate (Strakova et al., 2021), and presence of other microorganisms. According to several recent studies, the survival of *C. jejuni* can be enhanced in the presence of *Acanthamoeba* spp., which is an amoeba frequently found in water and soil (Siddiqui and Khan, 2012). It was previously shown that bacterial interactions with *Acanthamoeba* are a defense mechanism of various pathogens, e.g., *Legionella pneumophila*, *Vibrio* spp., *Pseudomonas aeruginosa*, *Listeria monocytogenes*, *Helicobacter pylori*, *Escherichia coli* and *Mycobacterium avium*, and may even act as a vector for transmission of these pathogens to hosts (Rowbotham, 1980; Cirillo et al., 1999; Winiecka-Krusnell et al., 2002; Reyes-Batlle et al., 2017). As for *C. jejuni*, cocultivation with the amoeba resulted in a delayed decline of bacterial numbers and a longer survival period at both sub-optimal temperatures and under AC (Axelsson-Olsson et al., 2005, 2010a,b; Baré et al., 2010; Bui et al., 2012a; Griekspoor et al., 2013).

It was previously reported that co-incubation with *Acanthamoeba* resuscitates *C. jejuni* cultures (Axelsson-Olsson et al., 2005, 2007; Reyes-Batlle et al., 2017), provides *C. jejuni* with increased acid resistance (Axelsson-Olsson et al., 2010a,b), protects it from chlorine and other chemical disinfectants (King et al., 1988; Snelling et al., 2005), and improves the survival of *C. jejuni* in milk and orange juice (Olofsson et al., 2015). However, the molecular mechanisms of interactions between *C. jejuni* and amoebas are not well understood, and the results of related publications are often conflicting. Some studies suggest *C. jejuni* adheres to the surface of the amoeba and survives extracellularly (Baré et al., 2010; Bui et al., 2012b), while others claim that *Campylobacter* cells enter the intracellular space of the amoeba, where it is able to survive and potentially multiply (Axelsson-Olsson et al., 2005, 2007, 2010b; Snelling et al., 2005; Griekspoor et al., 2013; Olofsson et al., 2013). The discrepancy could be partially explained by using various collection strains of *C. jejuni* and different strains of amoebae, which makes the results difficult to compare.

The increased aerotolerance of various *C. jejuni* isolates together with the prolonged survival of cells due to low temperatures and interactions with amoebas suggest that previously overlooked environmental niches might contribute to the transmission of *C. jejuni* and subsequently to the numbers of human infections. However, the majority of *Campylobacter*-related research solely uses isolates from food and clinical samples, whose phenotypic features might differ from the environmental isolates continuously stressed by low nutrition, low temperature, and a high concentration of oxygen. To fill this knowledge gap, this work focused specifically on the survival of several water isolates in comparison with one isolate that originated from a clinical sample, and one from raw chicken meat.

## Materials and Methods

### Bacterial Isolates and Culture Conditions

Experiments were performed with 12 *C. jejuni* water isolates, one isolate from raw chicken meat and one human clinical isolate (Table 1). All isolates have different genotype. The cultures were stored at −80°C in Brain-Heart Infusion medium (BHI; Oxoid, United Kingdom) containing 20% glycerol. At the beginning of each experiment, the isolates were resuscitated on Karmali agar plates (Oxoid, United Kingdom) at 42°C for 48 h under a microaerobic atmosphere (MAC; 5% O_2_, 10% CO_2_, 85% N_2_) in an MCO-18M multi-gas incubator (Sanyo, Japan).

**TABLE 1:** *Campylobacter jejuni* isolates used in the study.

**Isolate**	**Source**	**Year of isolation**
Cj5643P	Pond water	2019
Cj5648P	Pond water	2019
Cj5650P	Pond water	2019
Cj5653P	Pond water	2018
Cj5654P	Pond water	2019
Cj5683P	Pond water	2019
Cj5715P	Pond water	2019
Cj5623W	Outlet of a wastewater treatment plant	2019
Cj5629W	Outlet of a wastewater treatment plant	2019
Cj5640W	Outlet of a wastewater treatment plant	2019
Cj5689W	Outlet of a wastewater treatment plant	2019
Cj5716W	Outlet of a wastewater treatment plant	2018
Cj1M	Meat isolate	2019
Cj5718C	Clinical isolate	2019

### Antibiotic Sensitivity Testing

The susceptibility profiles of the isolates were determined by the standard disk diffusion method and evaluated according to the EUCAST guidelines using the most relevant and standardized antibiotics: ciprofloxacin (CIP; 5 μg/disk), erythromycin (ERY; 15 μg/disk) and tetracycline (TET; 30 μg/disk). Briefly, resuscitated colonies were suspended in phosphate-buffered saline (PBS) to a density of 0.5 McFarland turbidity standard. Using a cotton swab, the suspensions were inoculated onto Mueller- Hinton agar supplemented with 5% defibrinated horse blood and 20 mg/L β-NAD (MH-F) (BioRad, United States) and incubated with antibiotic disks at 42°C for 24 h under microaerobic conditions. The inhibition zones were recorded and interpreted according to EUCAST for *Campylobacter* spp.

### Aerotolerance Test

To test whether the cells can grow under AC, a single colony of the resuscitated isolates was inoculated onto fresh Karmali agar and incubated for 240 h under MAC. Grown cells were then harvested and resuspended in PBS (Gibco, United States) and standardized to 3.0 McFarland turbidity standard. Then, 10 μl of the standardized bacterial suspensions were subsequently inoculated onto Karmali agar plates and incubated at 42°C under AC, for 24–96 h. The presence of visible *C. jejuni* colonies at the end of cultivation was interpreted as the capability to grow under AC. The experiment was repeated three times with technical triplicates.

### Acclimatization of *Campylobacter jejuni* Isolates to Atmospheric Level of Oxygen

To acclimatize the cells to AC, the resuscitated colony of each isolate was first inoculated on Karmali agar and cultivated at 42°C for 24 h under MAC. The isolates were then successively subcultured three times on fresh Karmali agar and incubated for 24 h under AC. All cultures were subjected to standard Gram staining prior to the next cultivation step to verify the absence of a potential contamination.

### Survival of Isolates at Low Temperature (7°C)

For the survival assay, the starting suspensions of *C. jejuni* isolates were prepared by harvesting colonies grown on a Karmali agar plate with Mueller-Hinton broth (MHB; Oxoid, United Kingdom) to a final optical density at 600 nm (OD_600_) of 0.8. Five-milliliter aliquots of the inoculum were placed in 15-ml round bottom polyethylene tubes, and they were incubated at 7°C, both under aerobic and microaerobic conditions. Samples taken after 0, 24, 48, 72, 192, 384, 576, and 768 h of incubation were serially 10-fold diluted in PBS and subsequently transferred onto fresh Karmali agar plates as 10 μl droplets in 3 technical replicates. The plates were incubated for 24 h at 42°C under MAC to determine the numbers of colony-forming units. The plate counts were expressed in CFU mL^–1^.

### Amoeba Infection Assay

The culture of *A. polyphaga* (strain Linc Ap-1) was routinely maintained at room temperature as a monolayer in 25 cm^2^ tissue culture flasks containing 7 mL of the Proteose Peptone-Yeast Extract-Glucose medium (PYG; Greub and Raoult, 2002). The ability of bacteria to interact with *A. polyphaga* was observed by performing an invasion assay. Cultures of *A. polyphaga* were harvested by removing the liquid from the flasks and replacing it with fresh PYG. Flasks were then incubated in a freezer (−20°C) for 10 min to detach amoebae from the bottom of the flask (Axelsson-Olsson et al., 2007).

Prior to the assay, the susceptibility of *C. jejuni* isolates to gentamicin was tested independently by incubating overnight cultures of bacteria with 200 μg/mL. The bacterial colonies were resuspended in PYG, adjusted to an OD_600_ of 0.5, added to each well with gentamicin, and incubated for 2 h under MAC. The number of viable cells was evaluated by plate counting as described above.

Co-cultures of *C. jejuni* with monolayers of amoeba cells were performed in 12-well tissue plates. In the control well, PYG medium was used instead of the amoeba. Bacterial cells were harvested from overnight Karmali plates, suspended in PYG medium, and adjusted to an OD_600_ of 0.5 ± 0.1, which corresponds to approximately 5 CFU/ml × 10^7^ CFU/ml. Amoebae were enumerated using a Bürker chamber, diluted and seeded at a density of 100:1 *C. jejuni*: *A. polyphaga* per well.

To allow invasion to occur, co-cultures were incubated for 3 h at 25°C under an aerobic atmosphere to mimic the environmental conditions. This allows the cells to settle and form monolayers at the bottom of the wells. All samples were subsequently washed three times with PBS to remove non-associated bacteria. Each well was then treated with gentamicin (200 μg/mL) and incubated for 2 h at 30°C to eliminate any remaining extracellular and adhered *C. jejuni*. After the incubation with gentamicin, amoebae were washed three times with PBS to remove residual antibiotics. Finally, amoebae were lysed by adding Triton X-100 (final concentration 0.3%) for 15 min at room temperature to release intracellular *C. jejuni*. All samples were then serially diluted in PBS, plated onto a Karmali agar plate and incubated for 24–48 h at 42°C under microaerobic conditions to evaluate *C. jejuni* internalization. All experiments were carried out in three independent experiments using technical triplicates. Co-cultures were observed with an inverted light microscope (40× lens) to visualize the bacteria within amoebae.

## Data Analysis

All experiments were performed in three independent biological replicates. Viable counts were log_10_ transformed for further analysis. Calculations and graphs were processed with Microsoft Excel 2016.

## Results

### Antibiotic Sensitivity Testing

Isolates of *C. jejuni* collected from different water sources located all over the Czechia were subjected to the standard disk diffusion method according to EUCAST. Out of 14 isolates, 8 isolates were resistant to CIP, 5 isolates were resistant to TET, and one isolate was resistant to ERY. Five isolates were sensitive to all the tested antibiotics (Table 2). Interestingly, all the isolates originating from the wastewater treatment plants were resistant to at least one of the tested antibiotics, three out of five isolates were resistant to both CIP and TET. In contrast, isolates from pond water were mostly sensitive to all the tested antibiotics, except for the isolate Cj5683P, which was resistant to CIP, and Cj5650P, which was resistant to both CIP and ERY. Interestingly, TET resistance was only observed in isolates isolated from wastewater and the clinical sample.

**TABLE 2:** Antimicrobial resistance profiles of *Campylobacter jejuni* isolates.

**Antibiotic group**	**Antimicrobial agents (concentrations)**	**Resistant isolate No (%)**	**Isolates of *C. jejuni***
Fluoroquinolone	Ciprofloxacin (5 μg/disk)	8 (57.1%)	Cj5650P* Cj5623W* Cj5629W **Cj5640W** Cj5683P **Cj5689W** **Cj5718C** Cj1M
Tetracycline	Tetracycline (30 μg/disk)	5 (35.7%)	**Cj5623W** **Cj5640W** **Cj5689W** Cj5716W **Cj5718C**
Macrolides	Erythromycin (15 μg/disk)	1 (7.1%)	Cj5650P

*underlined, resistance to ciprofloxacin (CIP) and erythromycin (ERY); bold, resistance to CIP and tetracycline (TET).

### Development of Aerotolerance

As the isolates isolated from the surface waters had to withstand harsh environmental conditions, including the atmospheric concentration of oxygen, they were tested for their ability to develop aerotolerance. At first, their natural aerotolerance was tested by inoculating resuscitated cells on Karmali agar that was then incubated at 42°C directly under AC. The experiment was repeated three times. However, the results of the three independent experiments were inconsistent (Table 3). In the first experiment, 12 isolates were able to grow aerobically (nine within the first 24 h, two within 48 h, and one within 72 h of incubation). When the experiment was repeated for the second time, growth was only observed in 11 isolates – 7 isolates after 48 h and 4 isolates after 72 h. In the third repetition, growth was only observed in 4 isolates after 48 h of incubation.

**TABLE 3:** The ability of *Campylobacter jejuni* isolates to grow under aerobic conditions (AC).

**Independent experiment No.**	**Growth after aerobic cultivation**			**Total number of grown isolates**
	24 h	48 h	72 h	
1	Cj5643P Cj5648P Cj5650P Cj5653P Cj5683P Cj5715P Cj5716W Cj5623W Cj1M	Cj5640W Cj5689W	Cj5654P	12
2	No visible colonies	Cj5643P Cj5716W Cj5623W Cj5629W Cj5640W Cj1M Cj5718C	Cj5650P Cj5653P Cj5715P Cj5689W	11
3	No visible colonies	Cj5653P Cj5715P Cj5623W Cj5629W	No visible colonies	4

Due to the inconsistency of the data, the isolates were tested for their ability to adapt to AC and to develop aerotolerance. For this purpose, all isolates were subcultured once microaerobically to ensure a high fitness of the cells, and then cultivated in high numbers three times in a non-modified atmosphere. All the tested isolates were able to acclimatize and grow aerobically. To verify whether the acquired aerotolerance is permanent or temporary, acclimatized isolates were deep-frozen in BHI at −80°C for at least 72 h, and then directly cultivated aerobically. Growth was observed in 6 out of the 14 isolates (Cj5629W, Cj1M, Cj5650P, Cj5640W, Cj5716W, and Cj5648P), however, the cultivation time needed to develop visible colonies varied from 48 h up to 120 h.

### Survival at Low Temperature

**FIGURE 1:**
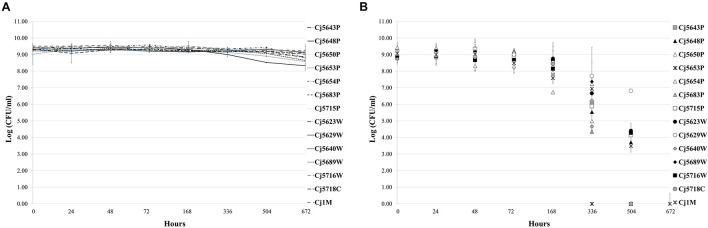
The survival of *Campylobacter jejuni* isolates under microaerobic **(A)** and aerobic **(B)** atmosphere at low temperature (7°C). Each point represents the mean calculated from three independent measurements.

Even though *C. jejuni* is generally thermophilic, the existence of isolates from relatively cold environments (such as surface water or chilled meat) suggests that it is able to adapt and survive at low temperatures. The isolates were therefore tested for their ability to survive cultivation in MHB at 7°C under both a microaerobic and an aerobic atmosphere. The viability of the isolates was monitored by regular plate counting over a period of 4 weeks. When cultivated under a MAC (Figure 1A), all the isolates remained viable over the 4 weeks with no significant decrease in viable cell numbers. In the aerobic cultivation (Figure 1B), the results were isolate dependent. In general, the numbers of cells slowly declined over the 4 weeks, up to a total loss of cultivability. As for particular isolates, one lost cultivability after 2 weeks of incubation (Cj5653P), 7 isolates lost cultivability after 3 weeks (Cj5643P, Cj5650P, Cj5654P, Cj5683P, Cj5689W, Cj5640W, and Cj5718C), and the remaining isolates were non-cultivable after 4 weeks of incubation.

### Protective Effect of *Acanthamoeba polyphaga*

To study the potential interactions with amoebae and follow the intracellular survival of *C. jejuni*, five isolates of *C. jejuni* were selected according to their ability to adhere and form biofilms (Shagieva et al., 2020). While suspensions of *C. jejuni* planktonic cells were completely inactivated by gentamicin, co-cultivation with amoebae resulted in an approx. 40% survival rate, irrespective of the tested isolate (Table 4). According to a microscopic observation, *C. jejuni* cells entered directly into the amoebae and were distributed as motile clusters within vacuoles.

**TABLE 4:** Survival rates of *Campylobacter jejuni* cells within *Acanthamoeba polyphaga*.

	**Starting suspension (log CFU/ml)**		**Gentamicin treatment**			
			*C. jejuni* + *A. polyphaga* (log CFU/ml)			*C. jejuni* (log CFU/ml)

**Isolate**	**Average**	**SD**	**Average**	**SD**	**Survival rate (%)**	
Cj5653P	7.71	0.004	3.28	0.051	34.73	No growth
Cj5654P	7.75	0.005	2.86	0.066	42.59	
Cj5716W	7.75	0.009	2.93	0.076	36.84	
Cj5623W	7.74	0.027	3.05	0.126	37.84	
Cj5718C	7.76	0.013	3.08	0.119	39.40	

*Intracellular survival rates were determined by colony-forming units (CFU) counted at 0 and 2 h post gentamicin treatment at 30°C under aerobic conditions (AC).*

## Discussion

As was mentioned above, water is an important source of human *Campylobacter* infections. An enhanced ability to survive in this low-nutrient, low-temperature, aerobic environment could be an important property, especially among isolates involved in water-related outbreaks. It can be expected that since it passes through various environments, *C. jejuni* overcomes harsh conditions, especially relatively high levels of oxygen and temperature swings, which can directly affect its viability. In this study, 14 isolates of *C. jejuni* were selected for further analyses, 12 of them being originally isolated from surface water in the Czechia. The general resilience of the isolates was evaluated by their antibiotic resistance, ability to survive and potentially grow under an aerobic atmosphere and at low temperature, and by their interactions with *Acanthamoeba polyphaga*. Recently, an increase in the resistance of *Campylobacter* spp. to antibiotics has been reported in several countries, including the Czechia (Serichantalergs et al., 2007; Bardon et al., 2009). Factors contributing to this increase could include the veterinary use of antibiotics as prophylaxis or for the treatment of animal diseases (Skandalis et al., 2021). In our study, more than half of the isolates (64.3%, 9/14) were resistant to at least one of the tested antibiotics, usually CIP. Interestingly, TET resistance was only observed in the isolates from wastewater treatment plants and from the clinical sample. This suggests that TET resistance was either acquired by the isolates in the wastewater treatment plant through horizontal gene transfer, or they originate from clinical cases and later reached the plant through the sewage systems. It is known that wastewater and sewage systems are a major reservoir of resistant bacterial populations due to the collection of antibiotic waste from humans and animals, improper disposal of drugs, and from factories, hospitals, and veterinary clinics (Nadimpalli et al., 2020). The resistance profiles of the waterborne *C. jejuni* suggest that these isolates may pose a significant public health threat, as quinolones (including CIP) are commonly used for campylobacteriosis treatment in clinical cases, especially the severe ones (Guk et al., 2019). In addition, there is evidence that patients infected with antimicrobial-resistant *Campylobacter* species have longer-lasting diarrhea than patients infected with antimicrobial-susceptible isolates (Nelson et al., 2004).

Despite the general perception that *C. jejuni* is microaerobic and therefore sensitive to oxygen, our data showed that *C. jejuni* isolates are able to survive and even multiply under AC. Some studies have already reported on the ability of *C. jejuni* isolates to survive in the presence of oxygen (Kaakoush et al., 2007; Oh et al., 2015a,b, 2017). However, those studies were mostly focused on the simple survival of the isolates in AC, not on their active growth. Recently, Rodrigues et al. (2015) characterized a unique human isolate of the *C. jejuni* strain, named Bf, that can grow under AC, suggesting that some isolates are highly resistant to aerobic stress. According to our results, the ability of aerobic growth is more common than expected, as all our waterborne isolates were able to adapt, develop aerotolerance and grow aerobically. Some of them were even able to maintain this ability even after short-term storage in the deep freezer. The microorganisms, once adapted, behaved as conventional aerobes and could be continuously maintained in aerobic subcultures for up to 3 days. In most isolates, aerobic growth was weak during the first 24 h, but improved with each subcultivation on fresh Karmali plates. It seems that aerotolerance is not a matter of different genetic background of the isolates, since the results of three independent direct cultivations under an aerobic atmosphere gave different results. Bronnec et al. (2016) have already sequenced one aerobic isolate of *C. jejuni*, but according to their results, the *C. jejuni* Bf gene repertoire required for resistance to oxidative stress did not differ from other isolates. As the aerotolerance was induced in all isolates, but was not retained for a long period of time, it is instead a matter of the regulation of gene expression. However, further research is needed to explain the regulatory mechanisms participating in the development of this aerotolerance.

The sensitivity to the concentration of oxygen goes hand in hand with the cultivation temperature. In many studies, it has already been mentioned that low temperature is an important factor affecting the survival of *C. jejuni* in an aqueous environment, including surface waters and sterilized water (Terzieva and McFeters, 1991; Buswell et al., 1998; Thomas et al., 1999; Tatchou-Nyamsi-König et al., 2007; Bronowski et al., 2014). To test whether the waterborne isolates are better adjusted to low temperatures than other isolates, they were cultivated at 7°C, both under aerobic and microaerobic conditions. All isolates were indeed able to survive at low temperature, which is consistent with previous observations that *C. jejuni* survives well under unacceptable temperatures for growth (Chynoweth et al., 1998; Chan et al., 2001; Bhaduri and Cottrell, 2004; Cole et al., 2004). However, their survival was more prolonged under a MAC than under air. All 14 isolates maintained their viability up to 4 weeks of microaerobic incubation without any strong decline in cell numbers, while under AC, the cultivability lasted for 1–3 weeks. In aqueous environments, the concentration of dissolved oxygen significantly varies between the different types of surface waters, as it is decreased by high pollution with organic substances, low flow rate, increased temperature, and even the presence of high numbers of competing microorganisms (Terzieva and McFeters, 1991). Thus, at least part of the aquatic environment could in fact be microaerobic. One of the studies demonstrated that *C. jejuni* populations survived longer in stationary than aerated water. Moreover, an increase in the cultivation temperature led to a decrease in the possibility of extracting viable *C. jejuni* (Rollins and Colwell, 1986). Similarly, Chynoweth et al. (1998) observed that isolates cultivated under microaerobic conditions in sterile stream water at 5°C survived better than when cultured aerobically. However, Buswell et al. (1998) showed that the degree of oxygenation affected survival in an unpredictable manner, and the effect was especially noticeable at lower temperatures. The survival of some isolates improved at lower oxygen levels, while it decreased for others.

Unlike under laboratory conditions, environmental water is full of other organisms that can directly affect the survival of *Campylobacter*, such as various protozoa. Therefore, selected waterborne isolates were investigated for their ability to interact with and be protected by *A. polyphaga*, as the protective effect of cocultivation with the amoebae had been previously suggested in several studies (Axelsson-Olsson et al., 2005, Axelsson-Olsson et al., 2010b; Snelling et al., 2005; Baré et al., 2010). According to our results, the cocultivation of all isolates for 3 h resulted in the survival of about 40% of cells treated with an otherwise lethal dose of gentamicin. The question remains of whether the numbers of protected cells were given by the capacity of amoebas and their vacuoles, or by the fact that within the 3 h of co-incubation some *C. jejuni* cells remained attached to the surface of the amoeba, and didn’t have enough time to enter the protective vacuole. On the whole, our results are in accordance with the results of Axelsson-Olsson et al. (2005), who demonstrated a prolonged intracellular survival of motile *C. jejuni* cells within amoebae, and with Vieira et al. (2017), who reported that the collection strain 81–176 can invade and survive inside *A. polyphaga* at 25°C for a certain period of time. However, it appears that not all *Acanthamoeba* species have the same effect. Our results conflict with a previous study by Dirks and Quinlan (2014), who showed that *C. jejuni* survives the assay independently of the presence of *A. castellanii*, and that the internalization of *C. jejuni* by *A. castellanii* is not consistent. Baré et al. (2010) also showed that co-culture with *A. castellanii* did not increase the numbers of either bacteria or amoebae, but delayed the decline and prolonged the survival of Campylobacter.

Interestingly, during the examined stress conditions, the water isolates of *C. jejuni* were resistant and survived at the same rate as the clinical and the meat isolate. However, no connection was found between the origin of the isolates and their particular features, except for the TET resistance observed in isolates from wastewater treatment plants and the clinical sample. In conclusion, this study proved that waterborne isolates of *C. jejuni* are able to adapt to different stress conditions that should supposedly impair their survival in the environment. As the resilience of the water isolates was comparable to both clinical and raw meat isolates, we suggest that water should be regarded as a significant reservoir of *C. jejuni*, and isolates of various water origins should be included in further research of this emerging pathogen.

## Data Availability Statement

The original contributions presented in the study are included in the article/supplementary material, further inquiries can be directed to the corresponding author.

## Author Contributions

ES and HM designed the experiments. ES performed the experiments and prepared the manuscript. KD and HM reviewed the manuscript. All authors contributed to the article and approved the submitted version.

## Conflict of Interest

The authors declare that the research was conducted in the absence of any commercial or financial relationships that could be construed as a potential conflict of interest.

## Publisher’s Note

All claims expressed in this article are solely those of the authors and do not necessarily represent those of their affiliated organizations, or those of the publisher, the editors and the reviewers. Any product that may be evaluated in this article, or claim that may be made by its manufacturer, is not guaranteed or endorsed by the publisher.
